# Psychometric behaviour of the strengths and difficulties questionnaire (SDQ) in the Spanish national health survey 2006

**DOI:** 10.1186/1471-244X-13-95

**Published:** 2013-03-22

**Authors:** Manuel Gómez-Beneyto, Andreu Nolasco, Joaquín Moncho, Pamela Pereyra-Zamora, Nayara Tamayo-Fonseca, Mikel Munarriz, José Salazar, Rafael Tabarés-Seisdedos, Manuel Girón

**Affiliations:** 1 Teaching Unit of Psychiatry and Psychological Medicine, Department of Medicine, University of Valencia, Valencia, Spain; 2 CIBERSAM, Instituto de Salud Carlos III, Madrid, Spain; 3 Research Unit for the Analysis of Mortality and Health Statistics, University of Alicante, Alicante, Spain; 4 Burriana Center of Mental Health, Burriana, Spain; 5 Paterna Center of Mental Health, Paterna, Spain; 6 CIBERSAM, Instituto de Salud Carlos III, Madrid, Spain; 7 Department of Clinical Medicine, Universitat Miguel Hernández, Sant Joan d’Alacant, Spain

**Keywords:** Psychometrics, Mental disorders diagnosed in childhood, Health survey, Strengths and difficulties questionnaire, Spain

## Abstract

**Background:**

The Strengths and Difficulties Questionnaire (SDQ) is a tool to measure the risk for mental disorders in children. The aim of this study is to describe the diagnostic efficiency and internal structure of the SDQ in the sample of children studied in the Spanish National Health Survey 2006.

**Methods:**

A representative sample of 6,773 children aged 4 to 15 years was studied. The data were obtained using the Minors Questionnaire in the Spanish National Health Survey 2006. The ROC curve was constructed and calculations made of the area under the curve, sensitivity, specificity and the Youden J indices. The factorial structure was studied using models of exploratory factorial analysis (EFA) and confirmatory factorial analysis (CFA).

**Results:**

The prevalence of behavioural disorders varied between 0.47% and 1.18% according to the requisites of the diagnostic definition. The area under the ROC curve varied from 0.84 to 0.91 according to the diagnosis. Factor models were cross-validated by means of two different random subsamples for EFA and CFA. An EFA suggested a three correlated factor model. CFA confirmed this model. A five-factor model according to EFA and the theoretical five-factor model described in the bibliography were also confirmed. The reliabilities of the factors of the different models were acceptable (>0.70, except for one factor with reliability 0.62).

**Conclusions:**

The diagnostic behaviour of the SDQ in the Spanish population is within the working limits described in other countries. According to the results obtained in this study, the diagnostic efficiency of the questionnaire is adequate to identify probable cases of psychiatric disorders in low prevalence populations. Regarding the factorial structure we found that both the five and the three factor models fit the data with acceptable goodness of fit indexes, the latter including an externalization and internalization dimension and perhaps a meaningful positive social dimension.

Accordingly, we recommend studying whether these differences depend on sociocultural factors or are, in fact, due to methodological questions.

## Background

In its 2006 edition, the Spanish National Health Survey (SNHS) used for the first time the Strengths and Difficulties Questionnaire (SDQ) to measure the risk for a mental disorder in children aged 4 to 15 years [1,2]. The SDQ provides separate scores for very important clinical and epidemiological dimensions, such as hyperactivity, emotional symptoms, behavioural problems and difficulties with peers. It also includes a prosocial behaviour, meant to measure the child positive social skills. In addition there are three versions to be used by the parents, the teachers and a self-report questionnaire for 11–16 16 years old, as well as an extended version which includes an estimation of the impact on functioning, distress and burden on others. This study will focus only on the parent version.

The SDQ was originally designed as a screening tool for population-based surveys [3,4] and it has been used in national health surveys in several countries [5-7]. It has also been used successfully for clinical evaluation in clinical settings and as a research tool. Studies undertaken in different cultures have shown that it possesses fair reliability and good criterion and convergent validity [8-10]. Regarding the internal structure there are a large number of studies confirming the existence of the aforementioned five theoretical dimensions, using both exploratory (EFA) and confirmatory (CFA) factor analysis [10,11]. However there are also discrepancies, some authors reporting three [12-15] and four factor solutions [16], and a few others who could not even find a clinically meaningful solution. A recent British study has confirmed both the five factor and the three factor solution [15] and in a study covering five European countries it is argued that the number of factors in the model may be country-dependent [14]. The three factor solution validated in some studies is particularly interesting since it gathers hyperactivity and behavioural problems in one factor, emotional symptoms and difficulties with peers in another factor and prosocial behaviour as a third factor. The first two factors constitute the well known dimensions of externalization and internalization. This is compatible with a hierarchical model of psychopathology. However the value of the prosocial behaviour dimension is not so clear. In constructing the questionnaire Goodman [17] added ten items reflecting traits of strength (half of them reverse-scored to reflect difficulties) to make it more acceptable to parents by enquiring about strengths as well as weaknesses. Out of ten strength items, the five directly-scored constitute the prosocial behaviour dimension, two reverse-scored items are included in hyperactivity, another two in peer problems and one in conduct problems. The introduction of strength items and directly/reversed-scored items has complicated the exploration of the factorial structure of the SDQ. A sixth factor including some of the strength items has been reported in previous studies and discarded as a methodological artefact [11,18].

The Spanish version of the SDQ [19] used in this study has been validated in a sample population of the Canary Isles [20] by a semi structured diagnostic interview [21] administered and scored by specialists. The diagnostic parameters obtained were acceptable and similar to those of the original study [3], but the cut point to identify probable cases was higher. Analysis of the dimensionality of the questionnaire using EFA showed a similar structure, though not equivalent to that expected from the theoretical structure.

The reported discrepancies in the structure of the SDQ and the uncertainties surrounding the Spanish version warrant a further examination of the psychometric behaviour of the SDQ. Thus, the aim of this study is to describe the diagnostic efficiency and internal structure of the SDQ in the sample of children studied in the SNHS 2006.

## Method

### Sample

The study data were obtained using the Minors Questionnaire of the SNHS 2006 [22]. The survey has a cross-sectional design, and contemplates a sample of children aged 0 to 15 years, distributed throughout Spain. Details of the methodology (sample design, sample size and sampling procedure) have been published elsewhere [2]. In brief, the number of children surveyed was 9,122, of whom 6,773 were aged 4 or over (51.2% men, 48.8% women). This latter was the size of the study sample, and was representative of the corresponding population. As a result of the complex sample design of the SNHS, the analysis used the weightings corresponding to the sample subjects. Applied to the children studied, these weightings enabled the number of children represented by each sample child to be established. The original weightings (λ) were calculated according to the sample design and included in the database supplied by the Spanish Ministry of Health, Social Politics and Equality, and were transformed to adjust the weights to the actual sample size studied. The estimations thus obtained were unbiased and coincide with those obtained using the methods incorporated in the sample design, although the random error of the estimations should be considered approximate. Among the various different solutions for use of the weightings we selected the method that consists of the transformation of the weightings under the normalized form:

Normalized weight sample unit i =

$$\omega =\frac{n}{N}\lambda_{i}$$

where n = number of sample minors

λ_i_ = original weight unit i.

N = population sample represented =

$$\sum_{i}\lambda_{i}$$

With these weights, a sample of the same size as that studied is reproduced, thus avoiding the problem of artificially reducing the random errors that would be estimated with the original weights, as these would reproduce a sample size similar to the study population, i.e., very large.

### Measures

To evaluate the presence of mental health problems, the survey included the Spanish version of the SDQ. The SDQ is composed of 25 questions grouped in five dimensions, four relating to psychopathology (emotional symptoms, conduct problems, symptoms of hyperactivity/inattention and peer problems) and prosocial behaviour. Each dimension has 5 items that are each scored between 0 and 2 according to their frequency, obtaining a score of 0–10 for each dimension. The total difficulty is obtained by adding the 20 items for difficulties (excluding prosocial behaviour).

The SNHS also includes questions directed to the informants of the child, aimed to detect cases of disease, with five of these questions being included in this study: Does the child suffer or has he/she ever suffered from conduct problems (including hyperactivity), Does the child suffer or has he/she ever suffered from mental disorder (depression, anxiety,…). If the answer to either of these two questions is “Yes”, then: Has he/she had them during the last 12 months? Has a physician ever said he/she has them? During the last 12 months, have these disorders or health problems limited the child in any of his/her usual activities in any way?

### Data Analysis

To estimate the efficiency of the questionnaire as a screening tool the ROC curve was drawn and calculations made of the area under the curve, sensitivity, specificity and the Youden J indices. The total difficulty score was used for the calculation and the children were considered to be cases if the informant had answered positively either of the first two questions and the remaining three designed to determine the presence of a disorder.

In order to estimate factor analysis models those cases in which a value was lost in any of the items studied were not included. The initial 6,773 minors fell to 6,506 who had complete information for all 25 items on the questionnaire. The factorial structure of the questionnaire was studied using models of exploratory factorial analysis and confirmatory factorial analysis, using the software FACTOR v8.1 [23,24] and LISREL v8.80 [25] respectively. The variables (items of the questionnaire) were defined as ordinal. The polychoric correlation matrixes between the items, obtained using the weightings corresponding to the sample subjects, were used as an element to reproduce both for the EFA and for the CFA.

In order to cross-validate factor models, the initial sample (n = 6,506) was divided in two random subsamples of the same size (n = 3,253). An EFA was performed on one subsample, using Parallel analysis based on 500 replications [26] as a test to establish the number of factors to retain. The estimation method was Unweighted Least Squares, and in order to obtain a simple factor solution we used Promin. This rotation method allows factors to be oblique in order to maximize factor simplicity [27]. The reliability of each of the factor construct was calculated after the model analyses.

The other subsample was used to validate the factor structure, previously obtained, by means of CFA. Four CFA models were adjusted. The first one with the factors obtained in the EFA (3 factors), without correlation structure. The second one with the same factors including their correlation structure. The third one with five factors obtained by EFA and with correlation structure. And the last one with the five-factor theoretical model described in the bibliography. As a general rule each item was assigned to an only factor, the one with the higher factor loading in EFA. The estimation method was Diagonally Weighted Least Squares. The goodness of fit of the CFA models was done with the usual indicators (Chi-Squared, RMSEA, ECVI, GFI, CFI, AGFI, NFI). Additionally, the reliability of each of the factor constructs was calculated in each factor as the proportion represented by the square sum of standardized factor loadings of its items with respect to the square sum of standardized factor loadings plus the sum of measurement errors associated with each item (McDonald's Omega index) [28].

## Results

The prevalence of conduct problems (including hyperactivity) diagnosed by a physician, present in the past 12 months and limiting activities of daily living, was 0.93% (CI: 0.70-1.16, n = 63), the prevalence of emotional symptoms (depression, anxiety) with the same characteristics was 0.47% (CI: 0.31-0.64, n = 32) and that of any disorder was 1.18% (CI: 0.92-1.44, n = 80). The area under the ROC curve for each of these diagnoses was 0.91 (CI: 0.88-0.94), 0.84 (CI: 0.77-0.91) and 0.88 (CI: 0.84-0.92), respectively. The diagnostic parameters for the presence of any disorder, for different cut points, are shown in Table 1.

**Table 1:** Diagnostic characteristics and Youden J index to diagnose the presence of any disorder

**SDQ cut point**^**a**^	**Sensitivity**	**Specificity**	**Youden J**
12.50	0,898	0,740	0,638
13.50	0,833	0,787	0,620
14.50	0,773	0,821	0,594
15.50	0,713	0,859	0,572
16.50 ^b^	0,652	0,887	0,539
17.50	0,648	0,905	0,553
18.50	0,645	0,926	0,571
19.50 ^c^	0,613	0,941	0,554

^a^ Positive result if the score is ≥ at the specified cut point.

^b^ Cut point proposed by Rodriguez Hernández J [20].

^c^ Cut point proposed by Goodman [4].

Before performing the EFA on the first random subsample, we calculated the Barlett's sphericity test, which was significant (p < 0.00001), and the Kaiser-Meyer-Olkin measure was 0.855, and so the data showed a good sampling adequacy for the factor analysis. Results of Parallel analysis suggested a three-factor model, since these are the only ones that explained variability above the mean of random replications. The three-factor model (F1, F2, F3) explained 50.0% of variability (26.1%, 15.0% and 8.8% variability explained by the respective factors) and the rotated loading matrix of which is given in Table 2. Only 4 items had a factor loading above 0.30 in more than one factor, and so the interpretation of the factors is rather clear. The construct reliability of the factors proposed for the model was 0.825, 0.908 and 0.880 for the respective factors F1, F2 and F3. A five-factor model was also built with these data in order to have a model with the same number of factors than the original theoretical model described in the bibliography. This model explained 59.4% of variability, 26.1%, 15.0%, 8.8%, 4.9% and 4.5% variability explained by the respective factors. These factors corresponded to the 5 eigenvalues above 1. The construct reliability of the factors proposed for the model was 0.786, 0.832, 0.908, 0.743, and 0.810 for the respective factors F1, F2, F3, F4 and F5. The rotated loading matrix for this model is given in Table 3, and only 2 items had a factor loading above 0.30 in more than one factor.

**Table 2:** Rotated loading matrix^*^of EFA with 3 factors (n = 3,253)

**Nº**	**Item**	**F1**	**F2**	**F3**
**3**	**Somatic**	**0.472**	0.127	0.006
**6**	**Solitary**	**0.536**	−0.148	−0.101
**8**	**Worries**	**0.644**	0.025	−0.018
**13**	**Unhappy**	**0.716**	−0.093	0.057
**19**	**Bullied**	**0.619**	−0.026	0.126
**23**	**Adults**	**0.425**	0.021	0.107
**24**	**Fears**	**0.386**	0.140	0.189
**1**	**Considerate**	0.043	**0.633**	−0.197
**4**	**Shares**	0.020	**0.617**	−0.016
**9**	**Caring**	0.170	**0.793**	−0.014
**11**	**Good friend**	0.246	**−0.755**	−0.148
**14**	**Popular**	0.217	**−0.736**	−0.092
**17**	**Kind to kids**	−0.033	**0.797**	0.010
**20**	**Often volunteers to help**	0.167	**0.668**	−0.103
22	Steals	0.276	−0.292	0.211
**2**	**Restless**	−0.044	0.138	**0.798**
**5**	**Tempers**	0.166	0.064	**0.591**
**7**	**Obedient**	−0.149	**−0.470**	**0.503**
**10**	**Fidgety**	−0.049	0.106	**0.850**
**12**	**Fights**	0.245	−0.134	**0.408**
**15**	**Distractible**	0.146	0.099	**0.506**
**16**	**Clingy**	**0.349**	0.011	**0.384**
**18**	**Lies**	0.101	−0.082	**0.514**
**21**	**Thinks before acting**	−0.132	**−0.383**	**0.518**
**25**	**Persistent**	−0.011	**−0.380**	**0.455**

(*) Factor loadings above 0.30 in bold.

**Table 3:** Rotated loading matrix^*^of EFA with 5 factors (n = 3,253)

**Nº**	**Item**	**F1**	**F2**	**F3**	**F4**	**F5**
**12**	**Fights**	**0.443**	0.163	−0.124	0.143	0.007
**18**	**Lies**	**0.554**	−0.038	−0.012	0.080	0.145
**22**	**Steals**	**0.802**	−0.060	−0.189	−0.223	−0.081
**23**	**Adults**	**0.433**	0.229	0.092	−0.203	−0.016
**3**	**Somatic**	0.034	**0.473**	0.095	−0.015	−0.066
**6**	**Solitary**	0.025	**0.480**	−0.108	−0.274	0.063
**8**	**Worries**	−0.011	**0.679**	−0.024	−0.022	−0.085
**13**	**Unhappy**	0.019	**0.778**	−0.156	0.034	−0.089
**16**	**Clingy**	−0.191	**0.604**	−0.048	0.269	0.238
**19**	**Bullied**	**0.336**	**0.475**	0.026	−0.195	0.034
**24**	**Fears**	0.093	**0.402**	0.126	0.044	0.052
**1**	**Considerate**	−0.102	0.016	**0.627**	−0.103	−0.066
**4**	**Shares**	−0.069	0.033	**0.606**	0.003	0.010
**7**	**Obedient**	0.138	−0.065	**−0.445**	0.218	0.277
**9**	**Caring**	0.056	0.112	**0.802**	−0.072	−0.013
**11**	**Good friend**	0.082	0.186	**−0.752**	−0.116	−0.122
**14**	**Popular**	0.048	0.165	**−0.694**	−0.190	0.035
**17**	**Kind to kids**	−0.043	−0.053	**0.835**	−0.085	0.116
**20**	**Often volunteers to help**	0.133	0.072	**0.644**	−0.014	−0.211
**2**	**Restless**	0.231	0.099	0.104	**0.505**	0.257
**5**	**Tempers**	0.234	0.258	0.021	**0.380**	0.116
**10**	**Fidgety**	**0.305**	0.069	0.084	**0.500**	0.271
**15**	**Distractible**	0.101	0.162	0.262	−0.180	**0.675**
**21**	**Thinks before acting**	0.017	−0.036	−0.295	0.041	**0.548**
**25**	**Persistent**	−0.012	0.019	−0.217	−0.243	**0.790**

(*) Factor loadings above 0.30 in bold.

The CFA’s were performed on the second random subsample. Four different models were built. According to the results of the EFA, two three-factor models were first adjusted, one of them without correlation and the other one with correlation between the factors. Secondly, two five-factor models were built. The first one according to the result of the five-factor model of the EFA and the second one according to the theoretical structure of the questionnaire, each factor comprising 5 items, such that each of the items on the questionnaire was assigned to just one of the 5 latent factors (according to the 5 subscales on the questionnaire). The adjusted models included the possible correlation structure between the latent factors.

Table 4 shows that all the correlated factor models had good indices of goodness of fit.

**Table 4:** Goodness of fit indexes for CFA models built on a random subsample of data (n = 3,253)

**Factor model**	**Chi-Square (df)**	**RMSEA** **(IC 90%)**	**GFI**	**AGFI**	**CFI**	**NFI**	**ECVI**
**Three uncorrelated factors according EFA analysis**	2736.8	0.0758	0.880	0.859	0.963	0.959	0.872
	(275)	(0.0740–0.0775)					
							
**Three correlated factors according to the EFA analysis**	2572.1	0.0597	0.933	0.920	0.965	0.961	0.823
	(272)	(0.0580–0.0615)					
							
**Five correlated factors according to the EFA analysis**	2255.0	0.0589	0.948	0.937	0.970	0.966	0.730
	(265)	(0.0571–0.0608)					
							
**Five correlated factors according to the theoretical model**	2693.3	0.0571	0.927	0.911	0.963	0.959	0.865
	(265)	(0.0553–0.0589)					

**Figure 1:**
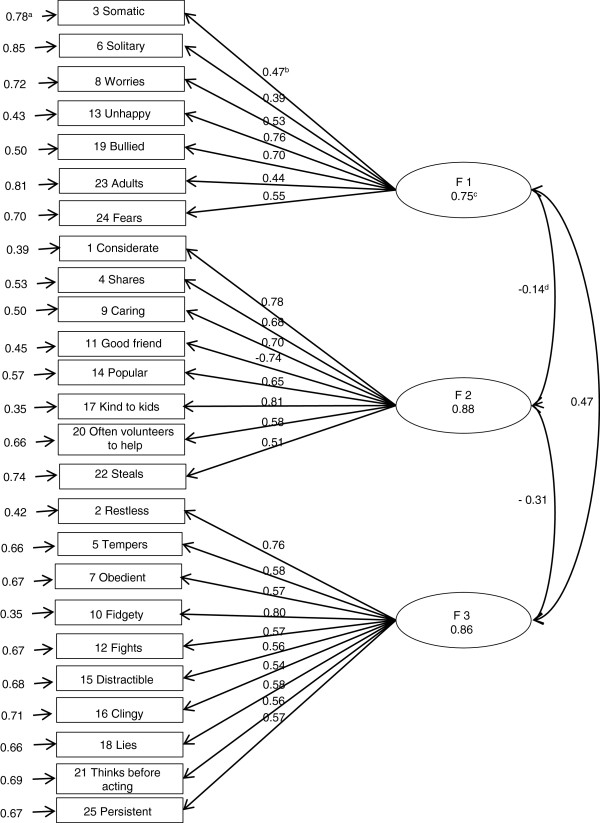
**Result of the CFA with 3 factors according EFA using the second random subsample (n = 3,253). a** Error term of variables; **b** Standardized factor loadings; **c** Reliability; **d** Correlation between factors.

**Figure 2:**
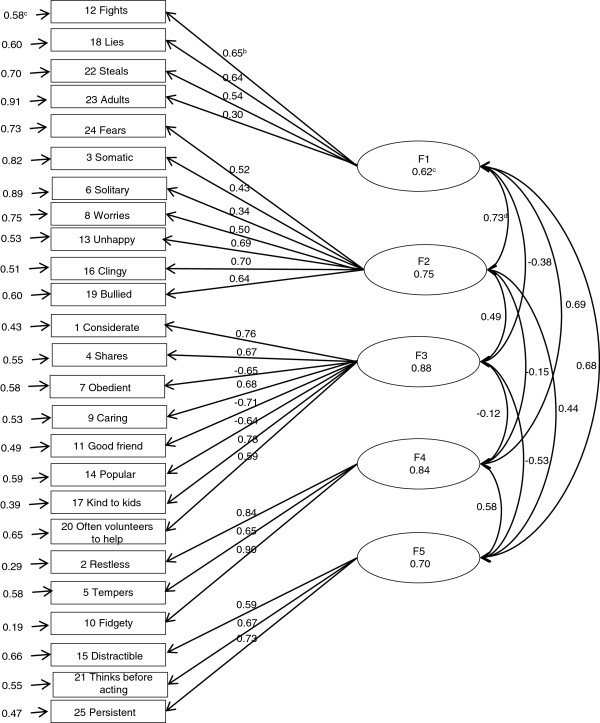
**Result of the CFA with 5 factors according EFA using the second random subsample (n = 3,253). a** Error term of variables; **b** Standardized factor loadings; **c** Reliability; **d** Correlation between factors.

**Figure 3:**
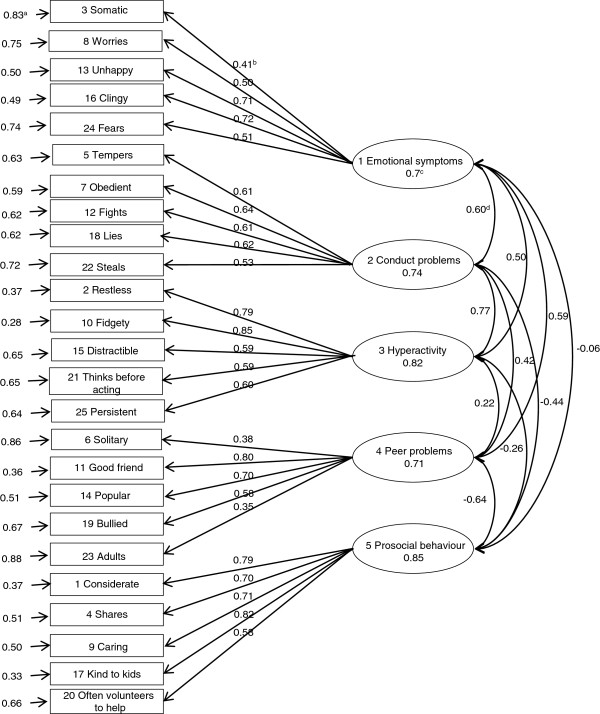
**Result of the CFA with 5 theoretical factors using the second random subsample (n = 3,253). a** Error term of variables; **b** Standardized factor loadings; **c** Reliability; **d** Correlation between factors.

Figures 1, 2 and 3 show the results of the standardized factor loadings, correlations between the factors, reliabilities of factors and error term of variables (items of the questionnaire) of the CFA models with correlated factors. All the standardized factor loadings were above 0.40 (except for item 6, which was 0.39 in the three-factor model and 0.34 in the five-factor model according to EFA, and for item 23, which was 0.30 in the five-factor model according to EFA.

The study of modification indices suggests the possible presence of some high correlations between certain items, which could improve the fit of CFA models.

## Discussion

The overall prevalence of cases detected in this study was 1.18%, being 0.93% for conduct problems (including hyperactivity) and 0.47% for emotional symptoms (depression, anxiety). These figures relate to the prevalence in minors who, according to their parents, had been diagnosed by a physician and who had also presented limitations in their activities of daily living during the previous 12 months; this prevalence of cases, therefore, was relatively severe. Considering that the prevalence of children clinically attended seen in this age range is around one sixth of that in the general population, the corresponding prevalence in the general population would be 5%, a value that agrees with that found in previous epidemiological studies in Spain [29].

The area under the ROC curve of 0.88 for the total difficulties is similar to the mean of 0.87 mentioned by Stone et al. [8] in a review of seven studies.

Considering the Youden J index as an indicator of the efficiency of the questionnaire [30], the cut point corresponding to the highest index (0.64) is 12/13, which indicates a sensitivity of 0.90 and a specificity of 0.74. This cut point is near that proposed by Goodman for the English population (15/16) and much below that proposed by Rodríguez for the population of the Canary Isles (19/20). Several earlier studies provide results on the sensitivity and specificity of the SDQ [8], but none of these surpasses those obtained in the present work.

All the factor reliability coefficients were acceptable (>0.70) except for factor 1 of the five factor solution (0.62). Reliabilities in previous studies are reported as Cronbach’s alpha and are generally low, particularly in conduct problems and problems with peers [8]. We have not found studies reporting on factor reliabilities. Concerning the use of Cronbach’s Alpha, unidimensionality of each scale is not entirely clear and the value of the Cronbach’s alpha could not be a good indicator of the internal consistency [31] and this why we use model-based reliabilities after the factor analysis.

Regarding the EFA three factor solution, only “steals” (item 22) loaded < 0.30. There were four items loading >0.30 in more than one factor (“obedient, item 7; clingy, item 16; thinks before acting, item 21 and persistent (item 25). CFA analysis in a different subsample confirmed the validity of this structure, including high factor reliabilities for the three factors.

The first and the third factor could be conceptualized as internalizing and externalizing dimensions respectively, and the third factor is clearly a social dimension. The internalizing dimension consists of four emotional symptoms plus “bullied” (item 19), “gets better with adults” (item 23) and “solitary” (item 6). This combination makes full sense from the clinical point of view. The externalizing dimension comprises all the hyperactivity and conduct problems plus “clingy” (item 16). This cluster is also clinically acceptable except for “clingy” which should belong to the internalizing factor.

The clustering of emotional symptoms under an internalizing dimension, and hyperactivity and behavioural problems under an externalizing one is in keeping with clinical and psychopathological knowledge and it has also been verified using other questionnaires. Thus, this is not a new finding but reinforces the validity of the SDQ by proving that it is in line with established psychopathological knowledge.

The second factor covers the five prosocial behaviour items, plus “good friend” (item 11) and “popular” (item 14), which in theory should belong to the peer problems dimension. These seven items constitute a meaningful combination of social items. However it should not be overlooked that three items which have higher factor loadings in the first and third factor, also load over 0.30 on this second factor. Taken together these ten items represent those added by Goodman to reflect strengths. Therefore it may well be that this factor is a method artefact, as noted by some authors [11]. Nevertheless, from our point of view there is not enough evidence to discard the social factor as an artefact. Prosocial behaviour and peer relationships are the bases of social capital and social capital plays an important role in social cohesion and in individual and public health [32]. Being such an important issue we think further research is warranted in establishing the validity of this dimension.

A three factor solution has been described in four previous studies [12-15]. Three of these studies reported a distribution of items identical to ours. However, Goodman [17] confirmed the validity of a somewhat different model: an internalizing dimension including all emotional and peer difficulties, an externalizing dimension including hyperactivity and conduct problems, and the prosocial behaviour.

In the EFA five factor model all items loaded >0.3 in only one factor, except for “bullied” (item 19) and “fidgety” (item 10). Factor reliabilities were acceptable. This model provides a solution similar to the theoretical structure originally proposed in several respects. The first and second factors include behavioural problems and emotional symptoms respectively as expected, except that “tempers” (item 5) is not included in behavioural problems and “bullied” and “solitary” (items 19 and 6) load in emotional symptoms. There are also important differences. “Good friend” and “popular” from the peer difficulties dimension (items 11 and 14) and prosocial behaviour cluster together, as in the three factor solution, making up again a meaningful social dimension. Finally the hyperactivity scale splits in two factors, hyperactivity and inattention, which is compatible with our current psychopathological understanding of Attention Deficit Hyperactivity Disorder. However, in spite of these discrepancies between the expected and the empirical model, the CFA confirmed the validity of the EFA five factor structure as well as the theoretical structure with good goodness of fit indexes.

The validity of the five factor model has been supported by the majority of previous SDQ studies. Those using EFA report different but closely similar distribution of items within the five factor structure. This is not surprising considering the different cultures where it has been tested and the use of parent, teacher or self-report questionnaires. Out of 18 studies reviewed by Stone [8], eight applied CFA and in five of them the five factor structure was supported using the parent version.

Finally, we may ask which of the two factor models is better. According to our estimations both models fit the data. Only two previous studies have also confirmed both models [14,15]. Whether these two models may have different applications in different circumstances or whether they reflect culture-dependent solutions is an open question. Goodman [15] gives some evidence to support the use of the externalization/internalization dimensions to screen for difficulties when surveying low prevalence populations. Essau [14] finds that the number of factors is dependent on the country where the survey has been carried out.

This study has some strengths and limitations. First, it is necessary to bear in mind the diagnostic criteria to define the result variable (case/non case) is very demanding, and it could not be comparable to a diagnostic interview.

The use of weightings corresponding to the sample subjects guarantee that the estimations are unbiased, although the random error could be underestimated. However, the size of the samples used both for the EFA and for the CFA was very high, and so we estimated models based on much evidence.

On the other hand, the use of polychoric correlation matrixes for the estimation of the factor analysis models resulted efficient and made it possible to incorporate both the weightings of the sample subjects and the ordinal metric of the items.

Even though the criteria to estimate CFA models was based on the assignment of each factor to the item with the highest factor loading in the corresponding EFA (three or five-factor models), the CFA made it possible to qualify the factor structures proposed as acceptable. The modification indices suggest the possible presence of some high correlations between certain items that could improve the fit of CFA models.

## Conclusions

The diagnostic behaviour of the SDQ in the Spanish population is within the working limits described in other countries. According to the results obtained in this study, the diagnostic efficiency of the questionnaire is adequate to identify probable cases of psychiatric disorders in low prevalence populations. Regarding the factorial structure we found that both the five and the three factor models fit the data with acceptable goodness of fit indexes, the latter including an externalization and internalization dimension and perhaps a meaningful positive social dimension.

Accordingly, we recommend studying whether these differences depend on sociocultural factors or are, in fact, due to methodological questions.

## Abbreviations

SDQ: Strengths and difficulties questionnaire; EFA: Exploratory factorial analysis; CFA: Confirmatory factorial analysis; SNHS: Spanish national health survey.

## Competing interests

The authors declare that they have no competing interests.

## Pre-publication history

The pre-publication history for this paper can be accessed here:

http://www.biomedcentral.com/1471-244X/13/95/prepub
